# Characterization of the Intraclonal Complexity of Chronic Lymphocytic Leukemia B Cells: Potential Influences of B-Cell Receptor Crosstalk with Other Stimuli

**DOI:** 10.3390/cancers15194706

**Published:** 2023-09-25

**Authors:** Andrea N. Mazzarello, Mark Fitch, Martina Cardillo, Anita Ng, Sabreen Bhuiya, Esha Sharma, Davide Bagnara, Jonathan E. Kolitz, Jacqueline C. Barrientos, Steven L. Allen, Kanti R. Rai, Joanna Rhodes, Marc K. Hellerstein, Nicholas Chiorazzi

**Affiliations:** 1 The Feinstein Institutes for Medical Research, Northwell Health, Manhasset, NY 11030, USA; 2 Department of Experimental Medicine, University of Genova, 16132 Genova, Italy; 3 Department of Nutritional Sciences & Toxicology, University of California at Berkeley, Berkeley, CA 94720, USA; 4 Zucker School of Medicine at Hofstra/Northwell, Hempstead, NY 11549, USA

**Keywords:** chronic lymphocytic leukemia, immunoglobulin, Toll-like receptor 9, intraclonal diversity

## Abstract

**Simple Summary:**

Chronic lymphocytic leukemia (CLL) clones contain cells differing in age: recently born, proliferative (PF), intermediate (IF), and resting (RF) fractions. We used deuterium incorporation into newly synthesized DNA in leukemic cells from patients with CLL to refine the “aging” kinetics, characterizing additional fractions differing in surface membrane (sm) CXCR4/CD5 levels, i.e., CXCR4^Dim^CD5^Dim^ double dim fraction (DDF) and CXCR4^Bright^CD5^Bright^ double bright fraction (DBF); and fractions differing in (sm)IgM and IgD densities. Although DDF was enriched in younger and DBF in older cells, PF and RF remained the youngest and oldest cells, respectively. Similarly, when using smIG to define subsets, cells with high smIgM and smIgD were the youngest, while cells with low smIgM and smIgD were the oldest. The youngest cells bore high levels of smIG and stimulating them via TLR9 and smIG yielded a phenotype that is more consistent with this in vivo observation. Finally, older cells were less sensitive to in vivo inhibition by ibrutinib. These data define additional CLL subpopulations; suggest that smIGs stimulation alone might not be responsible for the observed smIgM phenotype; and suggest that differential sensitivities of distinct fractions to the actions of ibrutinib might account, in part, for therapeutic relapse.

**Abstract:**

Chronic lymphocytic leukemia (CLL) clones contain subpopulations differing in time since the last cell division (“age”): recently born, proliferative (PF; CXCR4^Dim^CD5^Bright^), intermediate (IF; CXCR4^Int^CD5^Int^), and resting (RF; CXCR4^Bright^CD5^Dim^) fractions. Herein, we used deuterium (^2^H) incorporation into newly synthesized DNA in patients to refine the kinetics of CLL subpopulations by characterizing two additional CXCR4/CD5 fractions, i.e., double dim (DDF; CXCR4^Dim^CD5^Dim^) and double bright (DBF; CXCR4^Bright^CD5^Bright^); and intraclonal fractions differing in surface membrane (sm) IgM and IgD densities. Although DDF was enriched in recently divided cells and DBF in older cells, PF and RF remained the most enriched in youngest and oldest cells, respectively. Similarly, smIgM^High^ and smIgD^High^ cells were the youngest, and smIgM^Low^ and smIgD^Low^ were the oldest, when using smIG levels as discriminator. Surprisingly, the cells closest to the last stimulatory event bore high levels of smIG, and stimulating via TLR9 and smIG yielded a phenotype more consistent with the in vivo setting. Finally, older cells were less sensitive to in vivo inhibition by ibrutinib. Collectively, these data define additional intraclonal subpopulations with divergent ages and phenotypes and suggest that BCR engagement alone is not responsible for the smIG levels found in vivo, and the differential sensitivity of distinct fractions to ibrutinib might account, in part, for therapeutic relapse.

## 1. Introduction

Chronic lymphocytic leukemia (CLL) clones can be divided into three subsets based on time since last cell division: cells that are the most recently born (hence the “youngest”), that divided earlier (therefore “older”), and the furthest from the last cell division (the “oldest”) [1,2]. Understanding the dynamic changes of these cells and the unique functional properties at each phase might uncover novel therapeutic targets since each cell within a clone appears to traverse these three stages. In addition, higher clonal proliferation rates are associated with more aggressive clinical courses, presumably because dividing cells can develop new genome-wide mutations during DNA replication that cell division requires [3]. Thus, being able to target and eliminate the most recently divided cells could abort leukemia progression and the development of treatment resistance [4].

An approach to studying the intraclonal kinetics of CLL B cells in vivo is to label dividing cells by having patients drink deuterated “heavy” water (^2^H_2_O) and then determining the enrichment of ^2^H in the DNA of CLL cells [1]. Since ^2^H-DNA-bearing cells can be identified by the reciprocal surface densities of CXCR4 and CD5 [2], a 3-subpopulation model was developed which proposed that when CLL cells divide in tissue proliferation centers, they upregulate surface membrane (sm) CD5 levels and reduce smCXCR4 and various integrin/cell adhesion molecules [1,2]. The highest levels of the former mark cells that recently divided and the reduced levels of the latter allow cells to detach from the stroma and migrate into the blood. The young, robust CXCR4^Dim^CD5^Bright^ cells are referred to as the proliferative fraction (PF). Over time, plasma membrane levels of CXCR4 reappear, and levels of CD5 decrease as cells transition to the intermediate (IF; CXCR4^Int^CD5^Int^) and then to the resting (RF; CXCR4^Bright^CD5^Dim^) fractions [1,2]. Aging cells with higher smCXCR4 levels re-acquire the ability to migrate to the secondary lymphoid organs in response to CXL12/SDF1 and receive survival signals [5]. Although some of these could then be stimulated again and redivide, as occurs for memory B cells in lymphoid tissues, this process does not occur immediately for all RF cells [6]. However, our current understanding of these kinetic transitions does not consider all fractions based on CXCR4/CD5 expression and how they transition from one to another. For example, the model assumes a linear and concomitant transition of smCXCR4 and smCD5, although the CLL clone is composed of more intraclonal fractions than originally analyzed, and the stimulants required to generate the “youngest” CLL cells are not clearly defined.

In addition to expressing varying levels of smCXCR4 and smCD5, CLL cells co-express smIgM and smIgD, which play key roles in promoting CLL-cell survival and growth [7]. Indeed, the inhibition of signaling through the BCR is clinically beneficial, as evidenced by the effects of Bruton’s Tyrosine Kinase (BTK) inhibitors on the patient’s quality of life and disease progression [8,9]. Moreover, in normal B cells, smIgM and smIgD differ in membrane location and organization [10,11,12], the ability to bind multivalent antigens [11,13], and signaling sequelae when engaged independently [14]. Similar differences in signaling consequences between smIgM and smIgD occur in CLL B cells [15,16]. Indeed, the surface membrane levels and organization of IgM and IgD are differentially linked with leukemia progression and the ability to transduce intracellular signals upon engagement [16]. Moreover, several other receptors and cellular interactions can stimulate and activate CLL B cells, e.g., Toll-like receptors (TLRs) and CD40 [17,18,19,20], and modify cell stimulation, e.g., CD19 [12,21]. Thus, recently divided cells have likely experienced a multifactorial crosstalk between the non-leukemic cells in the tumor microenvironment (TME) and the various receptors expressed by the leukemic cells [6,22].

Herein, we have expanded the examination CLL kinetics in vivo by analyzing two new phenotypic fractions, CXCR4^Dim^CD5^Dim^ (double dim fraction, DDF), and CXCR4^Bright^CD5^Bright^ (double bright fraction, DBF), as well as fractions differing in smIG density (smIG^High^ and smIG^Low^). Each has been evaluated for time since last division/age and transitioning to a resting state, relative levels of smIgM and smIgD, and the synergistic roles of different activation pathways and co-stimulatory CD19 in determining the CLL phenotypes and kinetics observed. Moreover, the changes occurring in various fractions after treating patients with ibrutinib were evaluated.

## 2. Materials and Methods

### 2.1. Study Design

We used cryopreserved samples from previously untreated patients with CLL who participated in a clinical study designed to evaluate the effects of ibrutinib therapy on in vivo CLL kinetics [23]. These patients had ingested ^2^H_2_O for 4 weeks, thus allowing us to measure the levels of ^2^H-DNA in CD5^+^CD19^+^ cells as an indication of leukemic B-cell birth rates in vivo [23]. Based on the reciprocal co-expression of smCXCR4 and smCD5, 3 previously reported (CXCR4^Dim^CD5^Bright^, CXCR4^Int^CD5^Int^, and CXCR4^Bright^CD5^Dim^) and 2 additional, novel (CXCR4^Dim^CD5^Dim^ and CXCR4^Bright^CD5^Bright^) intraclonal fractions were sorted. These are referred to as the proliferative fraction (PF, CXCR4^Dim^CD5^Bright^), the intermediate fraction (IF, CXCR4^Int^CD5^Int^), the resting fraction (RF, CXCR4^Bright^CD5^Dim^), the double dim fraction (DDF, CXCR4^Dim^CD5^Dim^), and the double bright fraction (DBF, CXCR4^Bright^CD5^Bright^). The enrichment of ^2^H incorporated into DNA in the 5 fractions was determined and compared.

In addition, CLL B cells (CD5^+^CD19^+^) were sorted from the same cryopreserved cells into 3 fractions based on the densities of membrane sIgM and sIgD (smIgM^Dim^, smIgM^Int^, smIgM^Bright^, smIgD^Dim^, smIgD^Int^, and smIgD^Bright^). These fractions were also compared for their levels of ^2^H enrichment. Refer to the Supplementary Materials and Methods for further details [1,24].

Moreover, since our biologic and phenotypic findings relating to the PF were not consistent with the expected findings for cells that had recently experienced smIG engagement, the co-stimulation of anti-IGs through TLR9 and CD40 was assessed to evaluate their involvement in shaping the recently divided CLL phenotype. Although, in preliminary studies, TLR9 and CD40 gave similar results, the former stimulant appeared to induce more proliferation. Therefore, the co-stimulation through sIGs and TLR9 in various chronologic orders was carried out.

Finally, the intraclonal CXCR4/CD5 fractions from the same samples and their ibrutinib-treated counterparts were compared to identify the effects of this type of therapy on cell size and smIG densities, both measured by imaging flow cytometry (IFC) (Amnis ImageStream X MKII, EMD Millipore, Darmstadt, Germany). Refer to the Supplementary Materials and Methods for further details [16].

### 2.2. Patients and Samples

All patients provided written informed consent to use their samples [23]. The choice and number of the patients with CLL used were based on sample availability. The cohort included a total of 13 CLL patients. Deuterium incorporation and associated phenotypes for CXCR4/CD5 and sIG subpopulations were measured in 10 patients with CLL (Supplementary Table S1). Samples from 11 patients were used to evaluate phenotypic changes occurring during ibrutinib treatment; 8 of these patients were also analyzed for deuterium incorporation into DNA (Supplementary Table S1). For stimulation experiments, through TLR9 and BCR, a cohort of 32 CLL patients was used. The cohort was composed of cases with the following IGHV mutational status: 16 Mutated CLLs (M-CLLs), 15 Unmutated CLLs (U-CLLs), and 1 Mutated and Unmutated CLL (M-U-CLL).

### 2.3. Data Elaboration, Graphic Representation, and Statistical Analysis

Intraclonal variability for all parameters studied (e.g., IG densities and ^2^H-DNA) was evaluated concomitantly in two ways: [1] by comparing absolute values of various fractions (e.g., MFI and ^2^H-DNA), or [2] fraction change with respect to the IF for ^2^H-DNA incorporation and the CXCR4/CD5 phenotype or with respect to Int for mIg density, as in (X-IF or Int)/IF or Int with X = any fraction; in this way, Int = 0 and values > or < 0 for increased or decreased values, respectively. Data are presented as graph bars for all subpopulations. For CXCR4/CD5 and IGs intraclonal fractions, graph bars are paired with heatmaps, ranging from light blue (lowest value) to dark blue (highest value). Statistical analyses were performed using GraphPad software v7 and v8. For all comparisons, one-way ANOVA and Fisher’s LSD tests were used. The *p*-values are reported as follows: not significant, ns (*p* > 0.05); * *p* ≤ 0.05; ** *p* ≤ 0.01; *** *p* ≤ 0.001; and **** *p* ≤ 0.0001.

Information regarding antibodies, sample preparation, immunofluorescence, gating strategy, measurement of deuterium, and in vitro stimulation is available in the Supplementary Materials Methods section.

## 3. Results

**Figure 1:**
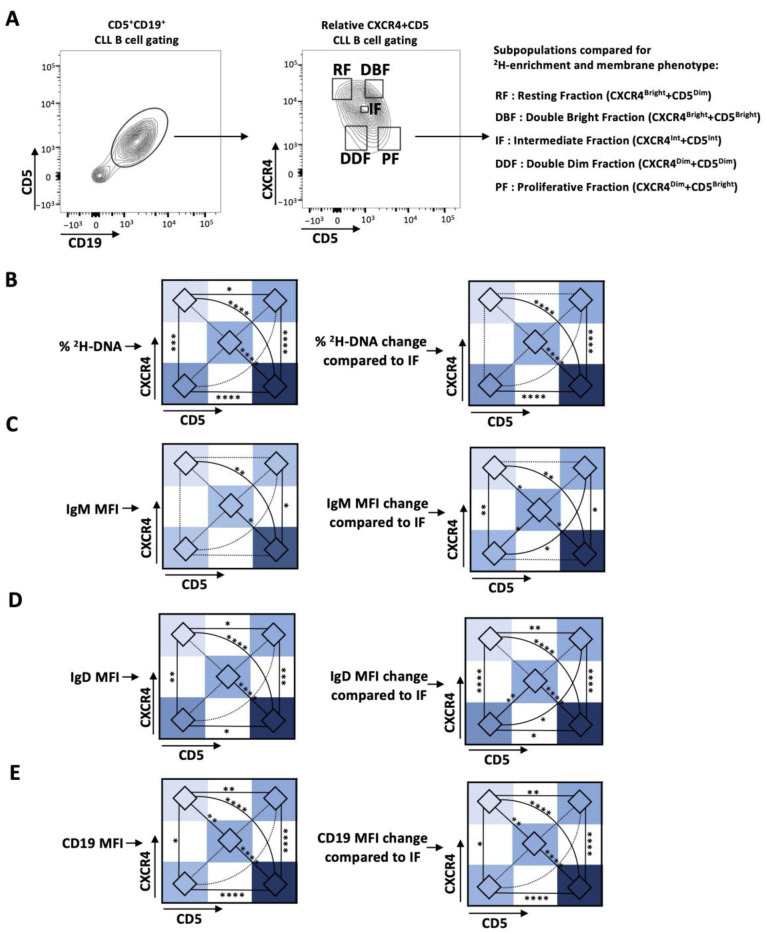
Deuterium (^2^H) enrichment in DNA and the IG and CD19 membrane levels of the CXCR4/CD5 intraclonal CLL subpopulations of patients with CLL. (**A**) Gating strategy for CXCR4 and CD5 relative density subpopulations. CD5^+^CD19^+^ B cells were separated into 5 subpopulations based on differences in the relative densities of membrane CXCR4 and CD5. Intraclonal comparisons in the subpopulations defined by CXCR4/CD5 of (**B**) levels of ^2^H-DNA enrichment; (**C**) IgM, (**D**) IgD, and (**E**) CD19 surface membrane densities. Data are presented as heatmaps showing the absolute percentage (**left**) and the relative levels as compared to that of the IF, represented as 0 (**right**). Nonsignificant *p*-value (*p* > 0.05) shown as dashed line. Significant *p*-values: * *p* ≤ 0.05, ** *p* ≤ 0.01, *** *p* ≤ 0.001, and **** *p* ≤ 0.0001.

In vivo kinetics of five CXCR4/CD5 intraclonal fractions: To dissect CLL intraclonal kinetics in greater detail, CD19^+^CD5^+^ cells from 10 patients with CLL were sorted based on the relative densities of CXCR4 and CD5 to define five fractions. These included the previously studied CXCR4^Dim^CD5^Bright^ (PF), CXCR4^Int^CD5^Int^ (IF), and CXCR4^Bright^CD5^Dim^ (RF) subpopulations, as well as two not previously characterized fractions expressing CXCR4^Dim^CD5^Dim^ (DDF) and CXCR4^Bright^CD5^Bright^ (DBF) membrane density levels (Figure 1A). The gating used was tailored for each case because the relative density pattern of CXCR4 and of CD5 is unique for each patient with CLL. In all cases, however, the IF is defined as the CXCR4 and CD5 intensity that the bulk of the clone displays (Supplementary Figure S1). See Material and Methods for a detailed explanation of the interpretation of these representations.

Consistent with previous reports, the CD5^+^CD19^+^ cells in the PF contained significantly higher enrichments in ^2^H-labeled DNA than cells of the RF and IF (Figure 1B and Supplementary Figure S2A). Notably, leukemic cells in the DDF contained significantly greater levels of ^2^H-labeled cells compared to the RF and significantly lower levels than the PF. Based on these findings, it was somewhat surprising that the levels of ^2^H-DNA^+^ cells were statistically similar between the DDF, IF, and DBF. This might reflect the number of cases studied and of the predominant enrichment in the PF compared to the other fractions. Consistent with these possibilities, the DDF contained more ^2^H-DNA-bearing cells than the IF (1.59-fold increase, *p* = 0.0617) and the DBF (1.47-fold increase, *p* = 0.1045) (Figure 1B and Supplementary Figure S2A). Moreover, the DBF contained significantly more ^2^H-DNA-bearing cells than the RF but significantly less than the PF (Figure 1B and Supplementary Figure S2A). Thus, the level of ^2^H-DNA enrichment in the five fractions is PF > DDF = IF = DBF > RF, with the PF containing the most cells temporally closest to the last cell division (the “youngest”), while cells in the RF are the farthest from the last cell division (the “oldest”). Moreover, using the level of ^2^H-DNA incorporation as an indicator of age, a unidirectional path of phenotype change would involve the youngest cells in the PF transitioning to the DDF or the IF or the DBF, with the latter three ultimately moving to the RF directly or indirectly through the IF.

Comparison of smIgM and smIgD densities during transition from the PF to the RF in the CXCR4/CD5 densities: BCR signaling is key in CLL pathogenesis, as evidenced by the effectiveness of drugs blocking this pathway [7,25]. Additionally, CLL smIgM and smIgD differ in their functional roles and clinical correlations [15,16]. Therefore, we measured intraclonal variability in smIgM and smIgD densities in the five CXCR4/CD5 fractions of interest. Cells with the highest levels of both smIgM and smIgD were found in the PF, and these were significantly greater than the IF, DBF, and RF. Additionally, the PF cells had a greater density than the DDF for IgD only, and smIgD densities differed the most between the DBF and the RF. Thus, smIgM densities follow this order, PF = DDF > IF = DBF = RF, whereas, for smIgD, PF > DDF > IF = DBF > RF (Figure 1C,D and Supplementary Figure S2B,C). In general, the distribution of different smIG densities resembles those seen for ^2^H-DNA incorporation (Figure 1 and Supplementary Figure S2).

Finally, CD19, a crucial co-stimulatory molecule in BCR-mediated cellular activation [21], was most and least expressed in the PF and RF, respectively, and similarly expressed in the DDF, IF, and DBF (PF > DDF = IF = DBF > RF) (Figure 1E and Supplementary Figure S2D).

**Figure 2:**
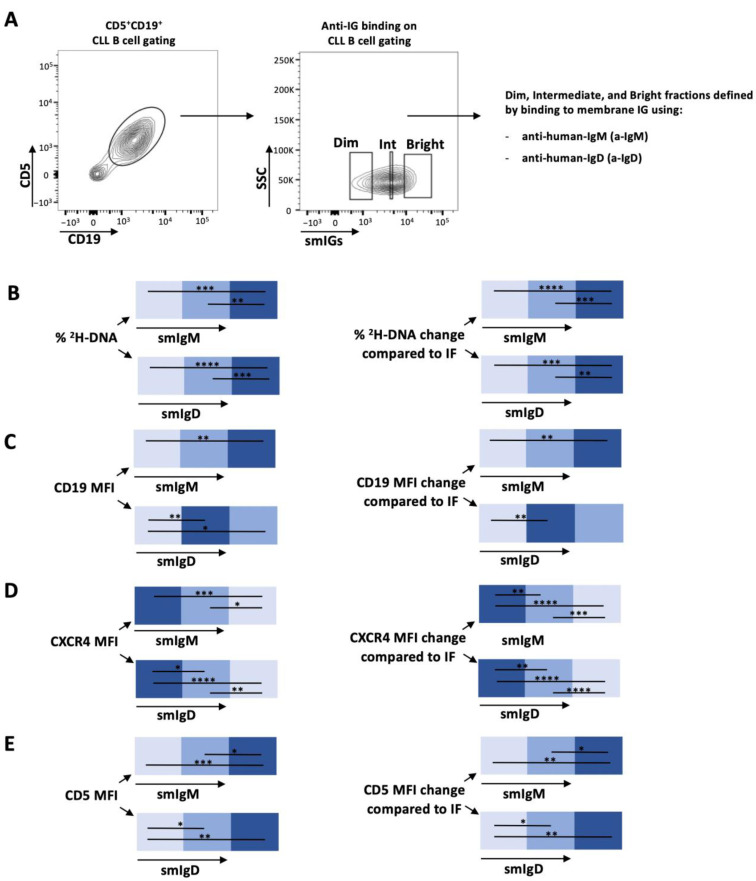
Deuterium (^2^H) incorporation into DNA of smIgM and smIgD intraclonal CLL subpopulations of patients with CLL who drank ^2^H_2_0 and the corresponding CD19, CXCR4, and CD5 membrane levels on those CLL cells. (**A**) Gating strategy for sIGs subpopulations. CD5^+^CD19^+^ cells were subcategorized based on dim, intermediate, and bright surface membrane densities of IgM or IgD. See Materials and Methods for details. Intraclonal comparison of (**B**) ^2^H-DNA enrichment and surface membrane levels of (**C**) CD19, (**D**) CXCR4, and (**E**) CD5. Data are presented as heatmaps showing the absolute percentages (**left**) or the relative changes with respect to the IF subpopulation (**right**) for subpopulations defined by smIgM (**top**) and smIgD (**bottom**) membrane densities. Nonsignificant *p*-value (*p* > 0.05) is shown as blank; significant *p*-value is reported as * *p* ≤ 0.05, ** *p* ≤ 0.01, *** *p* ≤ 0.001, and **** *p* ≤ 0.0001.

In vivo kinetics of the intraclonal fractions defined by smIgM and smIgD: Because of the similarity in the patterns of smIG density and ^2^H-DNA enrichments among the CXCR4/CD4 fractions, we next measured the enrichment levels of ^2^H-DNA-bearing cells differing in smIgM and of smIgD densities, using the MFI of soluble anti-IgM and anti-IgD binding as indicators (smIG^Dim^, smIG^Int^, and smIG^Bright^; Figure 2A). As performed for the CXCR4/CD5 density patterns (Supplementary Figure S1), IG gating was tailored for each case (Supplementary Figure S3). Comparing the enrichments in ^2^H-DNA-labeled cells in the smIgM and smIgD density defined fractions indicated that cells with the highest smIgM and smIgD levels had the highest ^2^H-DNA enrichment, and cells with the lowest smIgM and smIgD densities had the least (Figure 2B and Supplementary Figure S4A).

Next, we compared smCD19, smCXCR4, and smCD5. The CD19 levels in the various smIgM and smIgD density fractions increased directly from the smIG^Dim^ to the smIG^Bright^ (Dim < Int < Bright) fractions. However, only smIgD was significantly increased from the smIG^Dim^ to the smIG^Int^ fractions (Dim < Int = Bright). For CXCR4, the highest and lowest levels correlated with the lowest and highest smIG levels, respectively, whereas smCD5 densities followed the opposite pattern (Figure 2C–E and Supplementary Figure S4B–D). The CXCR4^Dim^CD5^Bright^ PF has the highest levels of smIgM and smIgD, and the CXCR4^Bright^CD5^Dim^ RF has the lowest.

**Figure 3:**
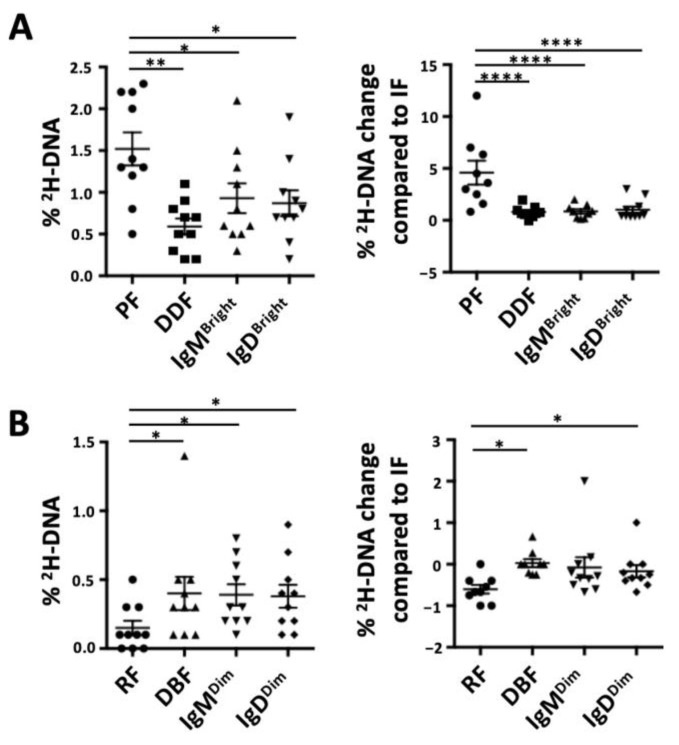
Comparison of the deuterium (^2^H) enrichment into DNA of intraclonal CLL subpopulations based on CXCR4/CD5 and on smIG density fractions. Graph bar for absolute percentages of ^2^H-DNA (**left**) or relative changes in ^2^H-DNA with respect to the IF/Int (assigned 0) are shown for (**A**) the most enriched subpopulations from each method of discrimination and (**B**) the least enriched subpopulations from each discrimination method. Nonsignificant *p*-value (*p* > 0.05) is shown as blank; significant *p*-value is reported as * *p* ≤ 0.05, ** *p* ≤ 0.01 and **** *p* ≤ 0.0001.

Finally, we compared the levels of ^2^H-DNA-labeled cells in the CXCR4/CD5 and the smIgM/smIgD intraclonal fractions. The CXCR4^Dim^CD5^Bright^ (PF) had the highest levels (Figure 3A), and the CXCR4^Dim^CD5^Bright^ (RF) had the lowest levels (Figure 3B), indicating that the CXCR4/CD5 density approach best identifies the cells in the blood that are closest and farthest from the last cell division.

The surface membrane phenotypes of the most recently divided CLL cells are not consistent with smIGs stimulation alone. Finding that the PF, which contains the circulating cells closest to the last cell division, has the highest levels of smIgM and smIgD is surprising since cell activation through the BCR is expected to lower at least smIgM levels [26,27], especially in CLL, where BCR signaling is critical [7,8,28,29]. Therefore, we tested if the engagement of other signaling pathways, whether alone or in combination with BCR engagement, would affect smIG densities on CLL cells.

In 32 patients, we measured smIG levels after stimulation through the TLR9 (CpG+IL15), CD40 (CD40L+IL-4), or BCR (anti-IgM or anti-IgD) pathways, individually or together (Supplementary Figure S5). Stimulation of CLL PBMCs with CpG+IL15 alone led to significant increases in smIgM (Supplementary Figure S5A), smIgD (Supplementary Figure S5B), CD19 (Supplementary Figure S5C), and CD5 (Supplementary Figure S5D); however, the changes for smCD19 were the greatest. Additionally, the cell size, which reflects cellular metabolic activity and is linked with CLL birth rates in vivo [16], was increased after TLR9 activation (Supplementary Figure S5E,F). Conversely, and as expected, selective engagement of smIgM or smIgD led to the downregulation of the corresponding isotype, with little or no effect on the other (Supplementary Figure S5A,B). Moreover, simultaneous engagement of smIgM and smIgD downregulated both isotypes (Supplementary Figure S5A,B). The CD19 and CD5 levels were also reduced (Supplementary Figure S5C,D). CD40 pathway activation led to similar, but less extensive, changes to those observed for TLR9. Therefore, only CpG+IL-15 stimulation was used for the following experiments.

Next, we tested if cells in the PF could be recent products of co-stimulation through the BCR and TLR9. Therefore, for 8 of the 32 patients studied above, we measured the smIgM, smIgD, smIgκ, smCD19, smCXCR4, smCD5 levels, as well as the cell size, after 5 days of individual or combined TLR9 and BCR stimulation delivered at culture initiation or at either BCR or TLR stimulation at initiation, followed by the other at Day 3.

**Figure 4:**
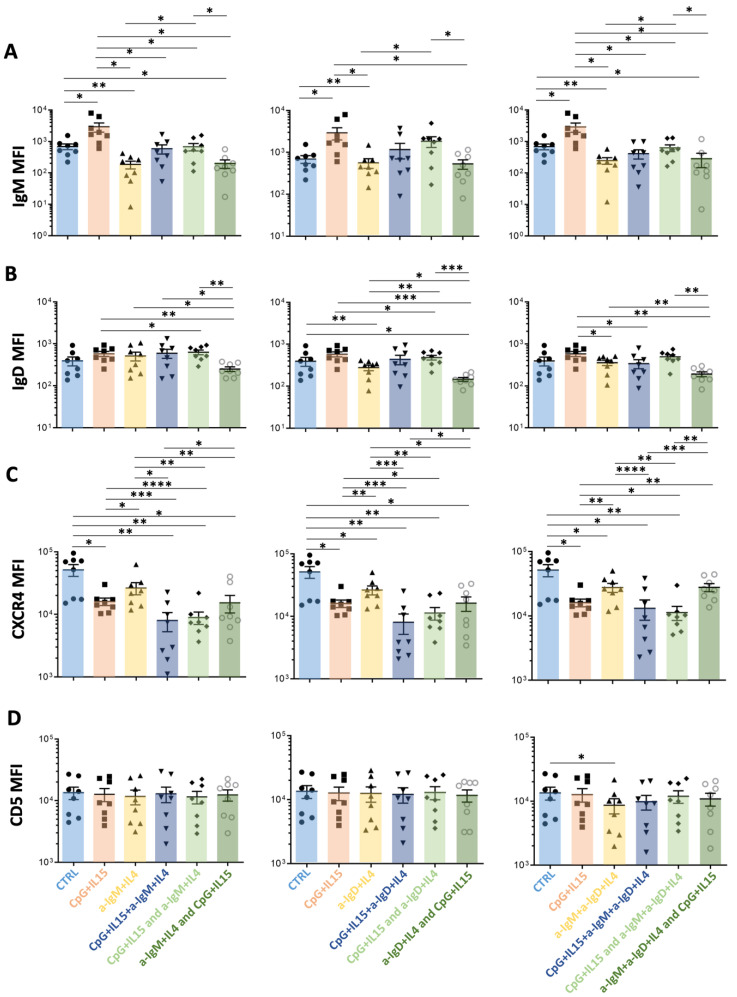
Comparisons of the effects on surface membrane amount of IgM, IgD, CXCR4, and CD5 on CLL cells after stimulation in vitro by TLR9 or anti-IGs, either alone or in combination, with the latter delivered at different time points. For each graph, the effects on MFI levels from the stimulation of the following receptors are represented: (i) control (Ctrl, light blue); (ii) TLR9 at day 0 (CpG+IL15, red); (iii) smIGs at day 0 (a-IGs+IL4, yellow); (iv) TLR9 and smIGs at day 0 (CpG+IL15+a-IGs+IL4, dark blue); (v) TLR9 at day 0 and smIGs at day 3 (CpG+IL15 and a-IGs+IL4, light green); and (vi) smIGs at day 0 and TLR9 at day 3 (a-IGs+IL4 and CpG+IL15, dark green). For each row, the TLR9 stimulation is represented in combination with anti-IgM (**left**), anti-IgD (**middle**), and anti-IgM + anti-IgD (**right**). The MFI changes in the various parameters were compared for the different stimulations for (**A**) IgM, (**B**) IgD, (**C**) CXCR4, and (**D**) CD5. Nonsignificant *p*-value (*p* > 0.05) is shown as blank; significant *p*-value is reported as * *p* ≤ 0.05, ** *p* ≤ 0.01, *** *p* ≤ 0.001, and **** *p* ≤ 0.0001.

Single TLR9 stimulation significantly upregulated smIgM density, while selective BCR stimulation significantly downregulated, below baseline, smIgM levels (Figure 4A). The increase in smIgM levels induced by CpG+IL-15 was inhibited and brought back to baseline by BCR engagement, delivered either concomitantly or up to 48 h after TLR9 stimulation (Figure 4A). However, when the BCR was engaged at 48 h prior to TLR stimulation, smIgM levels were reduced to those found after signaling through the BCR alone, which are less than baseline (Figure 4A). Thus, the enhancing effect of CpG stimulation was eliminated. Although less marked, effects such as those observed for smIgM were also found for smIgD (Figure 4B).

To ensure that the observed smIG changes were not caused by steric effects due to the binding of the anti-IgM and anti-IgD antibodies used for stimulation and staining, changes in the smIg light chain (kappa or lambda) were also measured (Supplementary Figure S6A). Notably, the smIgκ levels confirmed that the simultaneous stimulation through TLR9 and engagement of smIG aborts the increase in smIG seen with TLR9 stimulation alone.

In the same cultures, the smCD19 densities differed from those of smIgM and smIgD after TLR9 plus BCR engagement. Specifically, the CpG-augmented smCD19 levels remained significantly above the unstimulated levels and equal to the levels achieved by TLR9 engagement alone (Supplementary Figure S6B). Finally, engaging smIG led to decreased CXCR4 (Figure 4C), whereas only minor effects were observed for CD5 (Figure 4D).

Additionally, TLR9 activation alone, before, or concomitant with smIG engagement led to increased cell size measured by IFC (Supplementary Figure S6C,D). In contrast, the initial BCR engagement, followed by TLR9 activation, led to phenotypes resembling that of the stimulation through the smIGs alone for all described markers (Figure 4 and Supplementary Figure S6).

**Figure 5:**
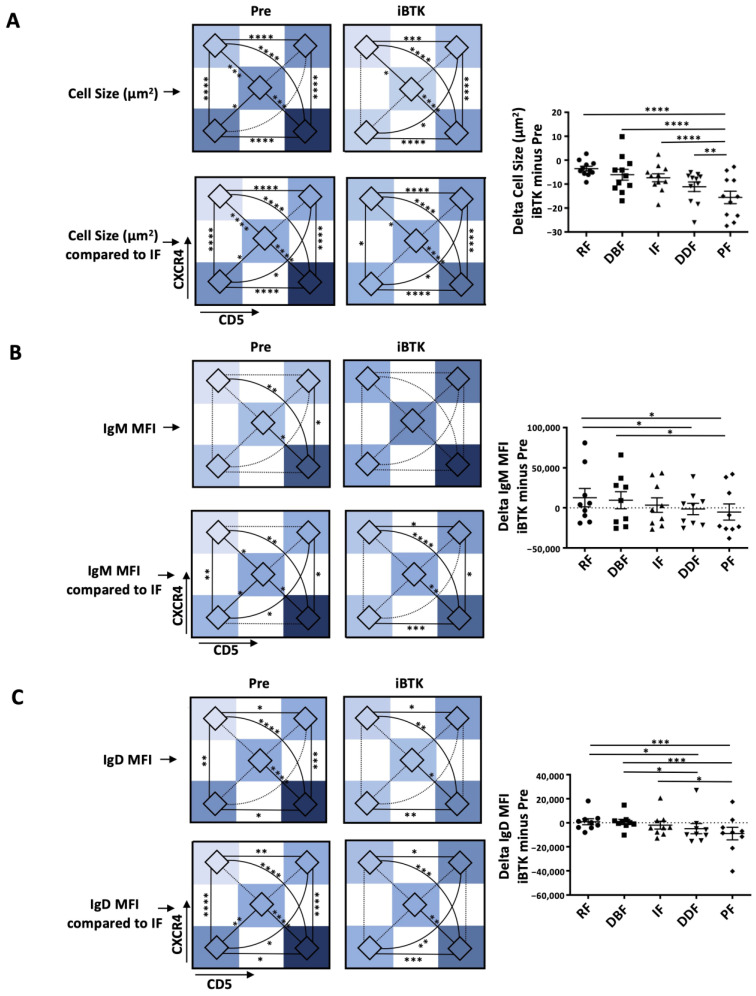
Changes in CLL cell size and smIG density of the CXCR4/CD5 intraclonal subpopulations during ibrutinib treatment in vivo. Measurements for CXCR4/CD5 intraclonal subpopulations before (Pre) and during in vivo ibrutinib treatment (iBTK) are represented as follows: (i) heatmaps of average area defined by imaging flow cytometry (IMF; **top**); (ii) heatmaps of area change with respect to the IF (assigned 0; **bottom**) and, delta change (iBTK minus Pre, **right**) for (**A**) cell size, (**B**) IgM, and (**C**) IgD. Delta measures are represented as bar graphs with mean ± SEM, and each dot represents one sample. Two outliers (highest and lowest value, respectively) were excluded. Nonsignificant *p*-value (*p* > 0.05) is shown as blank for histogram plots and as a dashed line for heatmaps; significant *p*-values are reported as * *p* ≤ 0.05, ** *p* ≤ 0.01, *** *p* ≤ 0.001, and **** *p* ≤ 0.0001.

Ibrutinib affects the intraclonal CXCR4/CD5 and smIG fractions differentially. The intraclonal patterns of time since last cell division and smIG densities were analyzed after BCR signaling inhibitor therapy in vivo by comparing smIG densities and cell size using IFC of cryopreserved samples taken from patients before and during ibrutinib treatment [23]. Before therapy, the intraclonal fractions containing cells of the largest size were those with greater ^2^H-DNA-enrichment and increased smIG levels (PF and DDF) (Figure 1B and Figure 5A; Supplementary Figures S2A and S7A). During ibrutinib treatment, the greatest changes in size occurred in cells of the PF and DDF (PF > DDF > IF > DBF > RF) (Figure 5A and Supplementary Figure S7A).

When the changes in smIG density during ibrutinib therapy were analyzed for the entire clone, there was a 1.35-fold increase in smIgM; however, this did not reach statistical significance. No differences were observed for smIgD density as previously reported Mazzarello et al. JCI 2022 [16]. Additionally, ibrutinib distinctively affected smIgM regulation, leading to significant smIgM changes between PF and RF, the fractions that, before treatment, displayed the highest and lowest smIgM (Figure 5B and Supplementary Figure S7B) and smIgD (Figure 5C and Supplementary Figure S7C) densities, respectively. Notably, the increases in smIgM were mostly due to the upregulation of IgM in the RF and DBF that are furthest from the last cell division (1.46-fold and 1.32-fold, respectively); this was associated, to a lesser extent, with a downregulation in the most recently divided fractions, PF (0.77-fold) and DDF (0.98-fold). Finally, smIgD was downregulated in the PF and DDF fractions (PF, 0.67-fold; DDF, 0.76-fold) but, in contrast to smIgM, was not upregulated in the RF and DBF fractions during treatment.

## 4. Conclusions

The addition of CXCR4/CD5 subpopulations (CXCR4^Dim^CD5^Dim^, DDF; and CXCR4^Bright^CD5^Bright^, DBF) to the previously studied subpopulations (CXCR4^Dim^CD5^Bright^, PF; CXCR4^Int^CD5^Int^, IF; and CXCR4^Bright^CD5^Dim^, RF) confirms that the PF and RF contain the youngest and oldest cells in CLL clones [1,2]. However, the additional findings indicate that not all intraclonal fractions, defined by differences in CXCR4/CD5 expression, follow a linear transition to the IF and RF as proposed in the initial model. Rather, the new findings imply that the most recently born cells enter the circulation as the PF from which they transition to either lower CD5 (DDF) or higher CXCR4 (IF and DBF) phenotypes, each eventually converging as the RF. However, we cannot exclude that CXCR4^Dim^CD5^Dim^ (DDF) cells enter the circulation directly, even though, based on ^2^H-DNA enrichment, these cells do not contain as many recently divided cells as the PF. Regardless, although the kinetics of the intraclonal CXCR4/CD5 CLL fractions are more complex than originally appreciated, there still appears to be a progressive change, starting from the PF (or the DDF) and ending in the RF.

In addition, we found that the fractions with the highest smIG densities (smIgM^Bright^ and smIgD^Bright^) contain more ^2^H-DNA-containing recently divided cells than the fractions with the lowest smIG density (smIgM^Dim^ and smIgD^Dim^). Hence, the youngest subpopulations are smIG^Bright^, while older, quiescent cells are smIG^Dim^. Additionally, when checking the CXCR4/CD5 fractions, the cells with the highest smIG densities are localized to the PF, confirming by another parameter that this fraction contains the highest levels of recently divided cells. Finally, CD19, which is essential for signal transduction through several receptors [21], was highest in the PF.

Remarkably, however, if cells in the PF were induced to divide solely by BCR engagement, finding high levels of smIgM and CD19 on these cells is unexpected since the engagement of BCR usually downregulates both receptors. Therefore, we asked if other stimulatory pathways could lead to smIgM^High^ and smCD19^High^ cells. Indeed, stimulation through TLR9 or CD40 led to increases in smIgM and smCD19 levels, with TLR9 being the more effective pathway and fitting the in vivo observations. However, when stimulation through TLR9 and the BCR was combined, the results were somewhat antagonistic. Specifically, stimulation of the BCR at the time of or up to 48 h after TLR9 engagement aborted the enhancement in smIG induced by TLR9 stimulation alone, but it did not lower smIG expression below the baseline level. However, the latter occurred when stimulating via the BCR alone or prior to TLR9 stimulation. Notably, the density of smCD19 was significantly increased despite BCR stimulation, regardless of the time of the smIG engagement.

Thus, if one were to speculate on how cellular stimulation leads to a smIgM^High^ phenotype in the most recently divided cells in vivo, TLR9 signaling could be involved. Such signaling could occur by circulating DNA molecules binding to the receptor for advanced glycosylation end products (RAGE) on CLL cells, directly or in a complex with HMGB1 [30], or binding of DNA or DNA-HMGB1 complexes to smTLR9 on the small circulating subpopulation of CLL cells, since both soluble DNA and HMGB1 are abundant in the sera of CLL patients [31,32], and the CLL cells bearing smTLR9 express the CXCR4^Dim^CD5^Bright^ phenotype of the PF [31]. In addition, intracellular TLR9 signaling could occur concomitant with BCR signaling [33,34,35]. For example, a poly/autoreactive CLL BCR could engage DNA, directly or as an immune complex with IG [36], and internalize and deliver DNA to TLR9, as happens in certain autoimmune settings [33,34]. Each model is supported by the increased expression of smCD19 that occurs upon the TLR9 activation of human B cells signaled through TLR9, as well as the BCR [37,38]. However, based on murine studies inactivating TLR signaling in vivo, the latter indirect method of DNA delivery might be more likely [39].

Regardless, when analyzing the intraclonal fractions in patients before and during ibrutinib therapy, cells of the largest size, comprising the most ^2^H-DNA-enriched cells with higher smIG levels (PF and DDF), showed the greatest changes in size and smIGs densities. This is consistent with BTK mediating complimentary actions of the BCR and TLR9 [40], and BTK inhibition affecting both BCR and TLR9 signaling [41]. Additionally, TLR9 and BCR signaling crosstalk might be linked to the variable effectiveness of ibrutinib on the various intraclonal fractions, having the greatest relative impact on CXCR4^Dim^CD5^Dim^smIGs^Bright^ and CXCR4^Dim^CD5^Bright^mIGs^Bright^ cells. The differential effects on each intraclonal subpopulation might explain the inability of BTKi therapies to achieve cures. Specifically, the subpopulations that are less affected by ibrutinib treatment (RF and DBF) might constitute a reservoir of leukemic cells that could divide again if appropriate stimulatory signals are delivered from the TME. This is consistent with ibrutinib and venetoclax targeting distinct intraclonal subpopulations (Figure 5, Supplementary Figure S7, and Lu et al. [42]) and with resistance to venetoclax occurring upon CD40L/TCR signaling [43,44,45,46].

In summary, our data define additional CXCR4/CD5 subpopulations of divergent ages, phenotypes, and sensitivities to BTK inhibition, suggesting that CLL B-cell kinetics are more complex than the current model describes. This complexity likely starts in secondary lymphoid organs, where serial engagements, possibly involving the BCR and TLR9, drive the generation of the PF that, once in the blood, continue their aging process toward the old/quiescent RF fraction. Although, at this point, our findings indicate that CXCR4/CD5 relative densities best identify the most recently divided CLL cells, more complex phenotypes involving CXCR4/CD5 relative densities with smIG densities might even better elucidate the subpopulations closets to cell division. Being able to identify and understand the underlying biological mechanisms that give rise to distinct intraclonal subpopulations, which differ according to membrane phenotype, biologic function, and susceptibility to therapies might permit the development of treatments that specifically target the multiple points in the CLL cell life cycle that all members of the clone appear to traverse.

## Data Availability

The data that support the findings of this study are available from the corresponding authors [A.N.M. and N.C.], upon reasonable request.

## Supplementary Materials

The following supporting information can be downloaded at https://www.mdpi.com/article/10.3390/cancers15194706/s1, Figure S1: CXCR4 and CD5 relative density patterns and gating positioning for each patient sample sorted and tested for deuterium (^2^H) enrichment in DNA in vivo; Figure S2: Distribution of deuterium (^2^H) enrichment in DNA of CXCR4/CD5 intraclonal fractions from patients with CLL who drank ^2^H_2_0 and the IG and CD19 membrane levels on those CLL cells; Figure S3: Relative surface membrane density patterns for IgM and IgD and gating positionings for each patient sample sorted and tested for ^2^H enrichment in DNA in vivo; Figure S4: Deuterium (^2^H) enrichment in DNA of smIgM and smIgD intraclonal fractions from patients with CLL who drank ^2^H_2_0 and the corresponding CD19, CXCR4 and CD5 membrane levels on those CLL cells; Figure S5: Effects on surface membrane phenotype of CLL cells upon selective engagement of TLR9 and/or IgM or IgD; Figure S6: Comparison of the effects on SSC-A, FSC-A, smIgκ, and CD19 by stimulation through TLR9 and smIGs, alone or in combination, with the latter delivered at different time points; Figure S7: Changes in CLL cell size and smIgM and smIgD densities on CXCR4/CD5 intraclonal subpopulations during ibrutinib treatment in vivo; Table S1: Clinical characteristics and laboratory features of the patients with CLL analyzed in this study.
