# Supplementary material for: Characterization of the Intraclonal Complexity of Chronic Lymphocytic Leukemia B Cells: Potential Influences of B-Cell Receptor Crosstalk with Other Stimuli
Source: Cancers (Basel). 2023 Sep 25;15(19):4706. doi: 10.3390/cancers15194706 (PMC10571896; doi:10.3390/cancers15194706)
Supplement: Supplementary file 1 [file cancers-15-04706-s001.zip › cancers-2588077-supplementary/cancers-2588077_Supp_Figure_and_Legend.pdf]

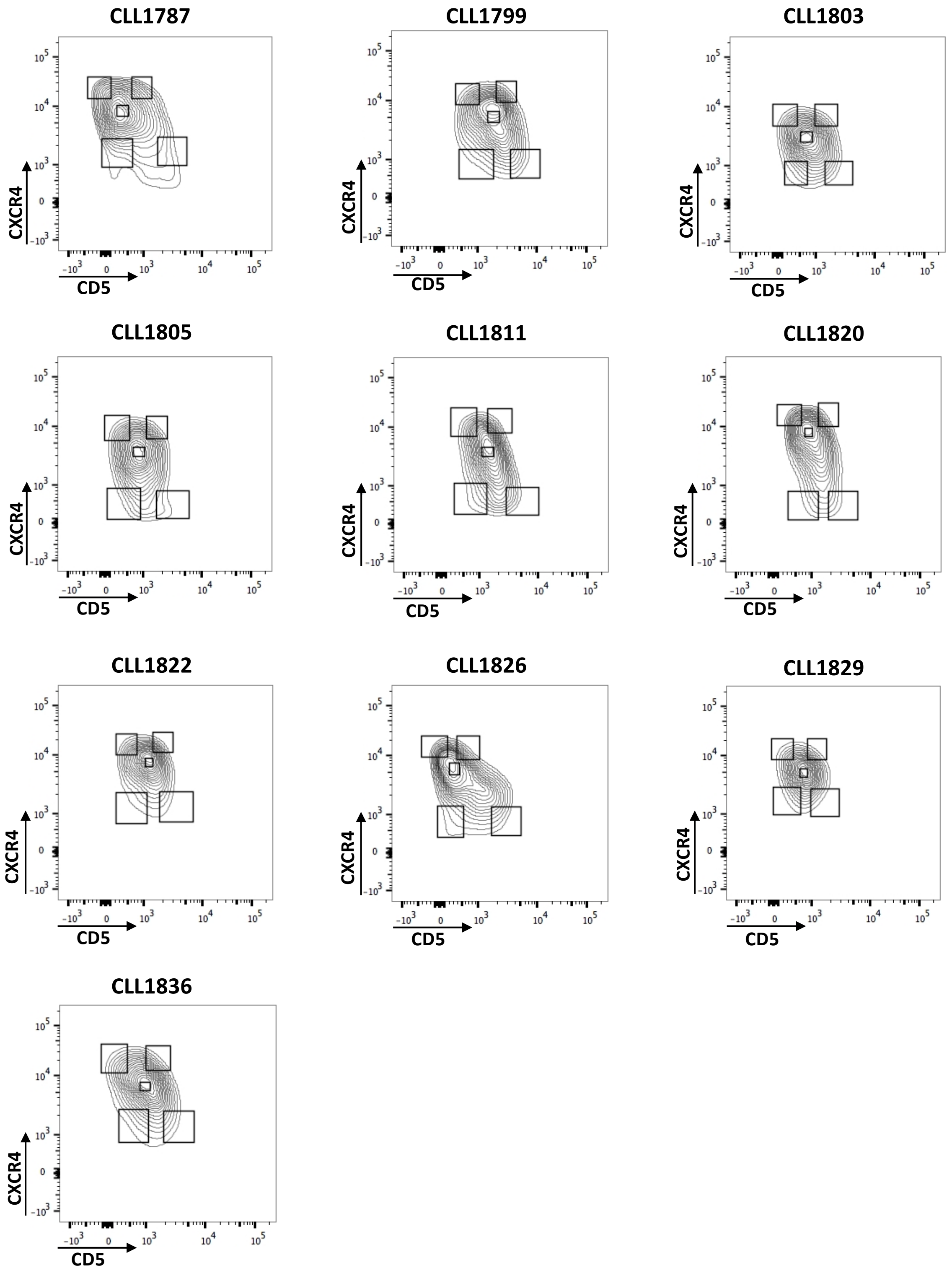

Figure S1. ***CXCR4 and CD5 relative density patterns and gating positioning for each patient sample sorted and tested for deuterium (^2H) enrichment in DNA in vivo.*** Cells bearing a

$CD5^+CD19^+$ surface membrane phenotype were plotted for CXCR4 and CD5. Because the relative patterns of the two markers can vary from patient to patient, the gates used to define the intraclonal subpopulations were adjusted according to each patient's sample. Gates define (from top left to bottom right) RF ($CXCR4^{Bright}CD5^{Dim}$), DBF ($CXCR4^{Bright}CD5^{Dim}$), IF ($CXCR4^{Int}CD5^{Int}$), DDF ($CXCR4^{Dim}CD5^{Dim}$), PF ($CXCR4^{Dim}CD5^{Bright}$) subpopulations.

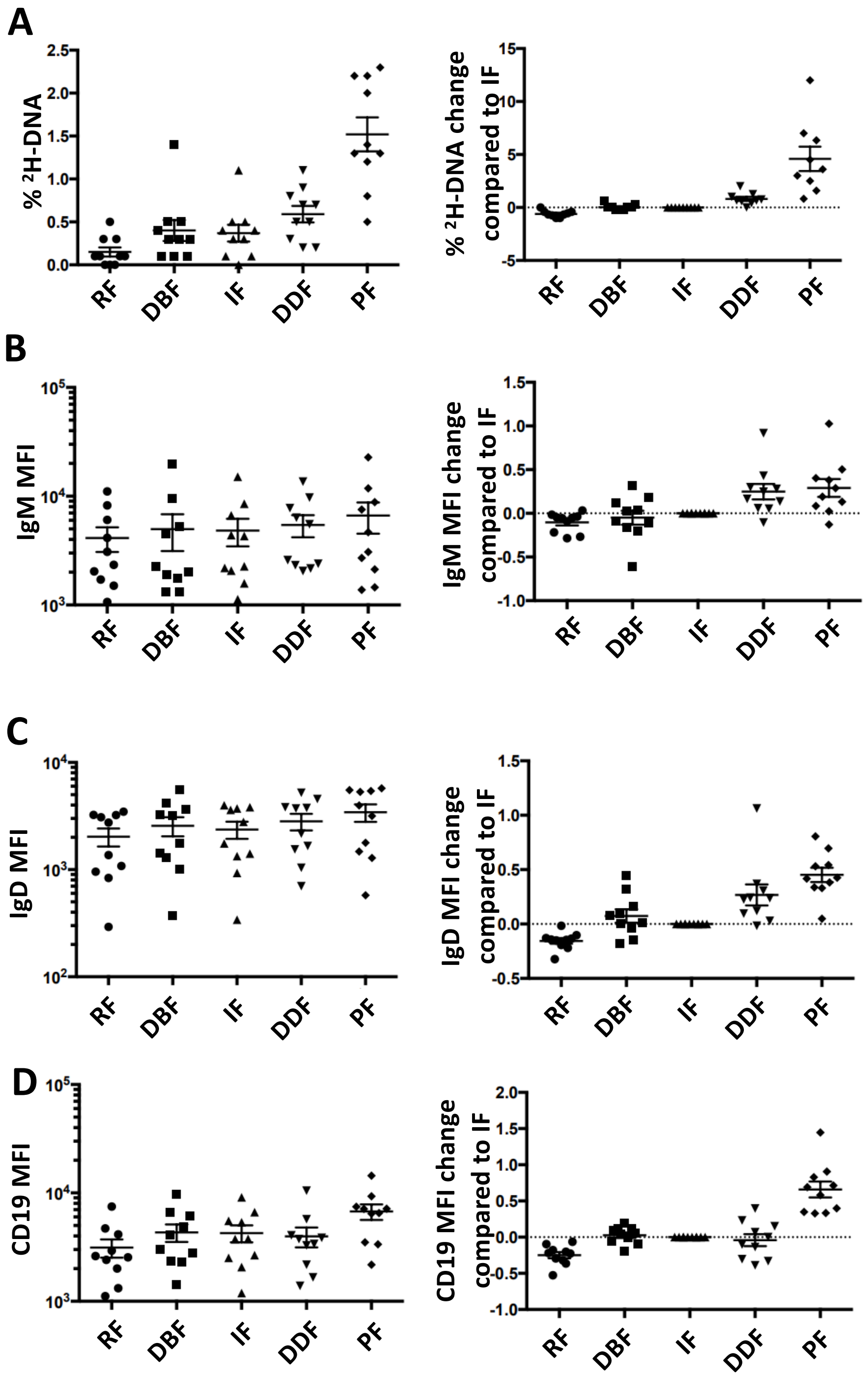

Figure S2. ***Distribution of deuterium (^{2}H) enrichment in DNA of CXCR4/CD5 intraclonal fractions from patients with CLL who drank $^2H_20$ and the IG and CD19 membrane levels on those CLL cells.***

The distributions of values reported in Figure 1 are shown, statistical values are the same as in

the main figure. Graph bar for absolute percentage and MFI (left) and the change in an individual parameter with respect to the IF (assigned 0; right) among the various CXCR4/CD5 subpopulations are reported for: **A**) ^{2}H-DNA enrichment in the 5 CXCR4/CD5 subpopulations, and the surface membrane levels, defined by MFI, of **B**) IgM; **C**) IgD and, **D**) CD19. Each dot denotes one sample. Bars represent mean $\pm$ SEM.

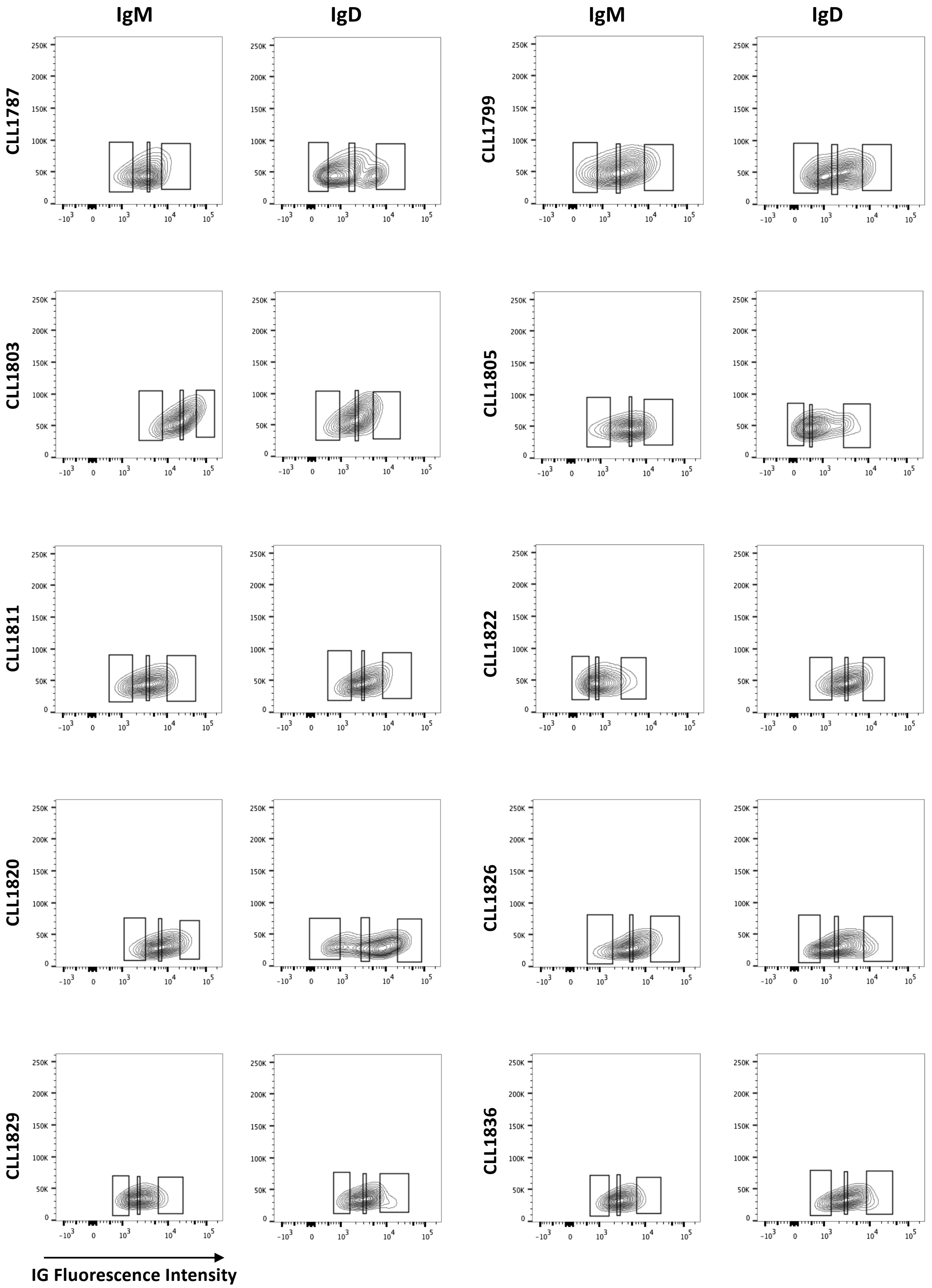

Figure S3. ***Relative surface membrane density patterns for IgM and IgD and gating positionings for each patient sample sorted and tested for ^{2}H enrichment in DNA in vivo.*** Because the average

IG surface membrane densities can vary from patient to patient, previously gated $CD5^+CD19^+$ cells were plotted for SSC-A (y axis) and FITC intensity (x axis) and intraclonal gates were adjusted to comprise ≤ 5% of the clone. Gates define dim, intermediate, and bright surface membrane densities of IgM and IgD based on binding of soluble anti-IgM (a-IgM) and soluble anti-IgD (a-IgD) Abs.

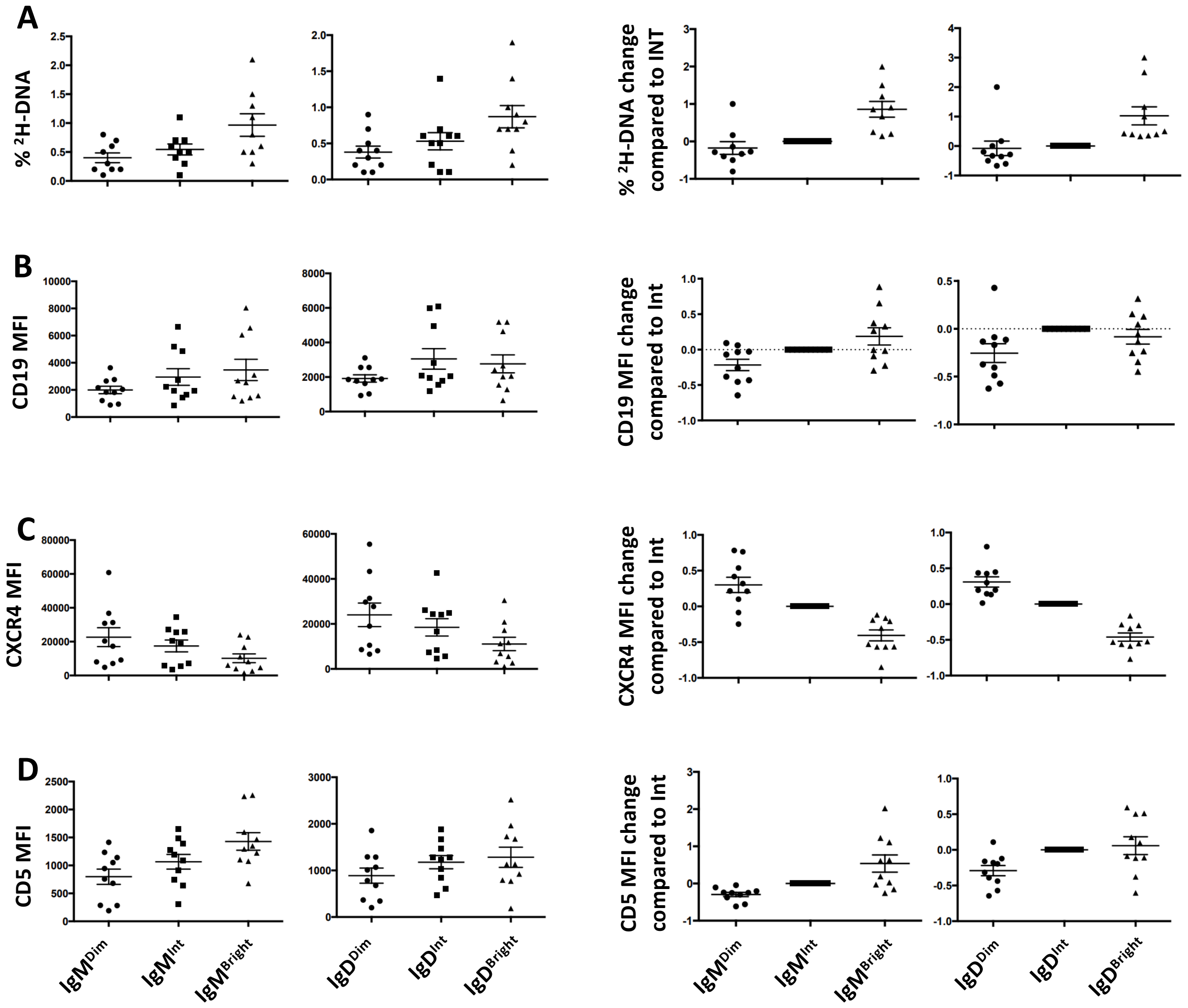

Figure S4. ***Deuterium (^{2}H) enrichment in DNA of* smIgM and smIgD intraclonal fractions from *patients with CLL who drank $^2H_20$ and the* corresponding CD19, CXCR4 and CD5 *membrane levels on those CLL cells*.** The distributions of values reported in Figure 2 are shown; statistical values are the same as for the main figure. Graph bar for absolute percentage and MFI (left) or the change with respect to the IF (right) among the various subpopulations is reported for: **A**) ^{2}H-DNA enrichment of the IG subpopulations based on surface membrane density; **B**) CD19; **C**) CXCR4, and **D**) CD5. Bars represent mean ± SEM. Each dot represents one sample.

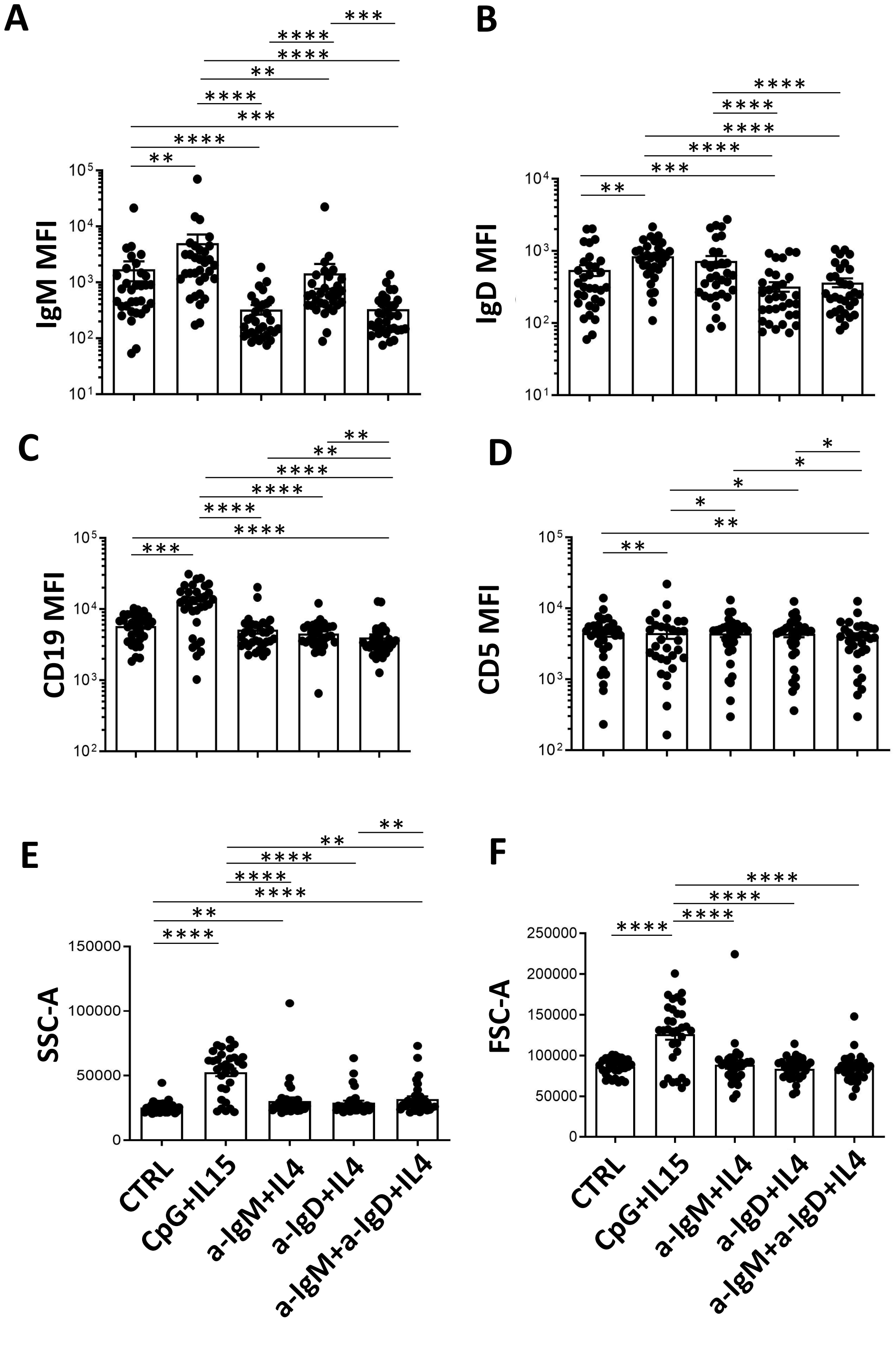

Figure S5. ***Effects on surface membrane phenotype of CLL cells upon selective engagement of TLR9 and/or IgM or IgD.*** N = 32 CLL cases were used (15 U-CLL, 16 M-CLL and 1 U/M-CLL). For

each graph, the following stimulations are represented (left to right): i) Control (Ctrl); ii) TLR9 (CpG+IL15); iii) smIgM (a-IgM+IL4); iv) smIgD (a-IgD+IL4) and; smIgM and smIgD (a-IgM+a-IgD+IL4). Changes in MFI after the various stimulations are shown for: **A**) IgM and IgD; **B**) CD19 and CD5; and **C**) SSC-A and FSC-A. Non-significant *P*-value ($P > 0.05$) is shown as blank; significant *P*-value is reported as: * $P \leq 0.05$; ** $P \leq 0.01$; *** $P \leq 0.001$; **** $P \leq 0.0001$.

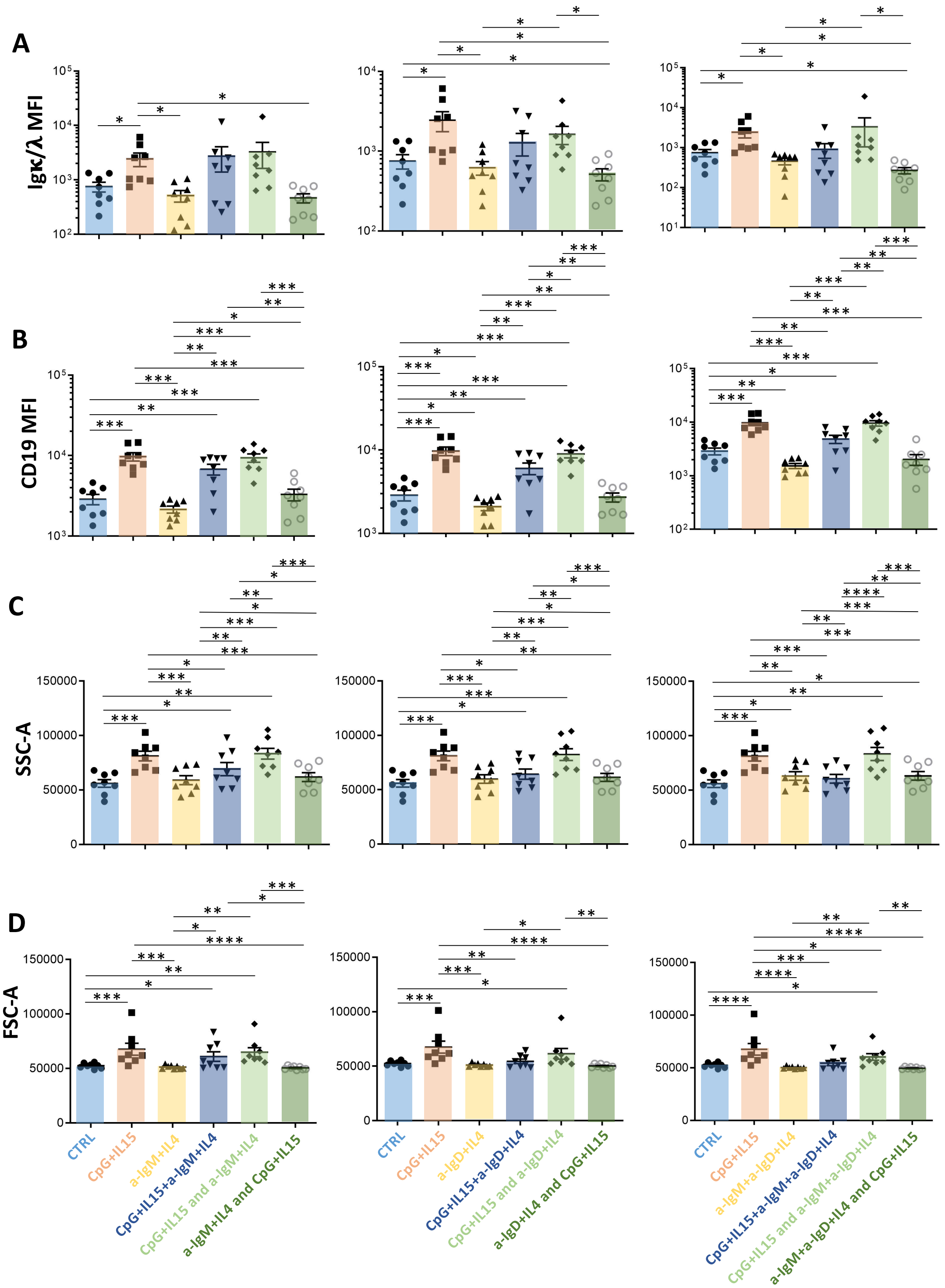

Figure S6. ***Comparison of the effects on SSC-A, FSC-A, smIgκ, CD19,*** **by** ***stimulation through TLR9 and smIGs, alone or in combination, with the latter delivered at different time points.*** For each

graph, stimulation of the following receptors are represented: i) Control (Ctrl); ii) TLR9 at day 0 (CpG+IL15); iii) smIGs at day 0 (a-IGs+IL4); iv) TLR9 and smIGs at day 0 (CpG+L15+a-IGs+IL4); v) TLR9 at day 0 and smIGs at day 3 (CpG+IL15 and a-IGs+IL4); vi) smIGs at day 0 and TLR9 at day 3 (a-IGs+IL4 and CpG+IL15). For each row, TLR9 stimulation is represented in combination with smIgM (left), smIgD (middle) and smIgM + smIgD (right). MFI changes were compared after the different stimulations for: **A**) mIgκ, mIgγ; **B**) CD19; **C**) SSC-A and **D**) FSC-A. Non-significant *P*-value ($P > 0.05$) is shown as blank; significant *P*-value is reported as: * $P \leq 0.05$; ** $P \leq 0.01$; *** $P \leq 0.001$; **** $P \leq 0.0001$.

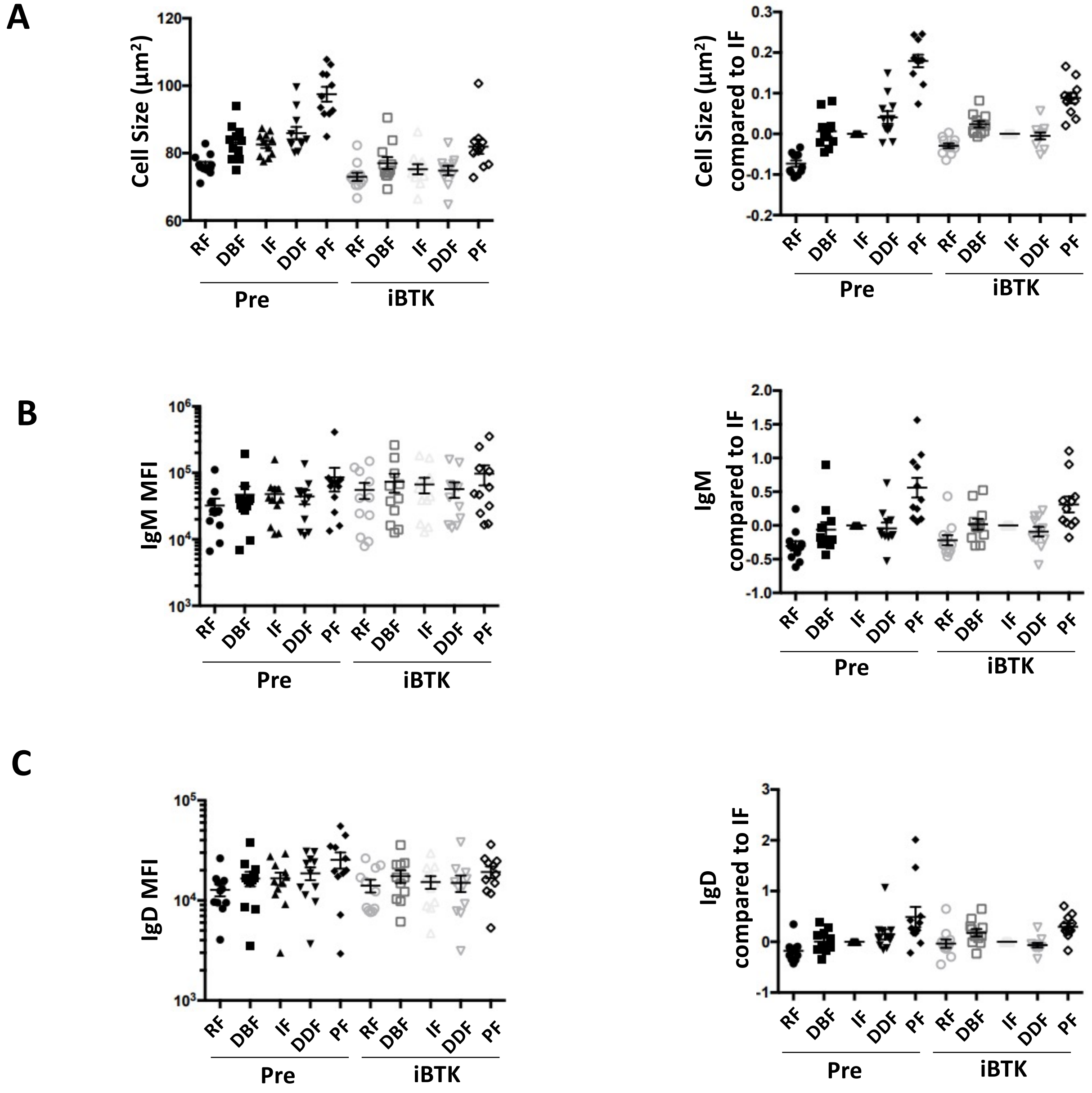

Figure S7. ***Changes in CLL cell size and smIgM and smIgD densities on CXCR4/CD5 intraclonal subpopulations during ibrutinib treatment in vivo.*** The distributions of values reported in Figure 5 are shown; statistical values are the same as the main figure. Measurement of the intraclonal CXCR4/CD5 subpopulations before (Pre) and during *in vivo* ibrutinib treatment (iBTK) are shown as average area or MFI, defined by IFC (left) or relative change in area or MFI with respect to the

IF (assigned 0; right) represented as bar graphs for: **A**) Cell size; **B**) IgM; and **C**) IgD. Bars represent mean $\pm$ SEM. Each dot represents one sample.

Table S1. ***Clinical characteristics and laboratory features of the patients with CLL analyzed in this study*.** For each patient, *IGHV* mutation status (U = *IGHV*-unmutated; M = *IGHV*-mutated); Rai stage; percentage of $ZAP70^+$ cells; percentage of $CD38^+$ cells; genetic abnormalities defined by fluorescent in situ hybridization; response to ibrutinib treatment (PR = partial response); use of sample for sorting and for intraclonal measurements of ^2H-enrichment in DNA; use of sample for comparison of intraclonal changes during treatment with ibrutinib.
