# Supplementary material for: Characterization of the Intraclonal Complexity of Chronic Lymphocytic Leukemia B Cells: Potential Influences of B-Cell Receptor Crosstalk with Other Stimuli
Source: Cancers (Basel). 2023 Sep 25;15(19):4706. doi: 10.3390/cancers15194706 (PMC10571896; doi:10.3390/cancers15194706)
Supplement: Supplementary file 1 [file cancers-15-04706-s001.zip › cancers-2588077-supplementary/cancers-2588077_Supp_Material_and_Methods.pdf]

Supplementary Materials and Methods

Antibodies. For flow cytometry (FC) and fluorescence-activated cell sorting (FACS), anti-hCXCR4-PE (R&D Systems, cat # Fab173P); anti-hCD5-PerCP-Cy5.5 (BD Biosciences, cat # 341089); anti-hCD19-Pacific Blue (eBioscience, cat # 48-0199-42); goat $F(ab')_2$ anti-IgM-FITC (Southern Biotech, cat # 2022-02); goat $F(ab')_2$ anti-IgD-FITC (Southern Biotech, cat # 2032-02) and goat $F(ab')_2$ IgG isotype control (Southern Biotech, cat # 0110-02) were used.

For IFC, the same antibodies listed above were used except for anti-hCXCR4-PECy7 (BioLegend, cat # 306514) and anti-hCD5-PE (BioLegend, cat # 300608).

Sample preparation and immunofluorescent staining for FC, FACS, and IFC. PBMCs from patients with CLL that were isolated by density gradient centrifugation with Ficoll-Paque Plus (GE Healthcare), cryopreserved in 10% DMSO and 90% FCS and stored in liquid nitrogen until use. To avoid membrane receptor capping and internalization, all procedures were carried out on ice using cells suspended in PBS with 0.02% NaN_3 and 1% FCS (staining buffer). Cells were aliquoted, incubated for 30 min with the appropriate mix of antibodies, washed 3 times with 200 µl staining buffer, and re-suspended with 100 µl BD Cytofix solution (BD Bioscience) for 1h. For phenotype measurements using FC and IFC, antibody mixes included anti-CD5, -CD19, -CXCR4, and one anti-IG (anti-IgM or anti-IgD) or isotype-matched control pAbs. To isolate CXCR4/CD5 fractions by FACS, each sample was stained with anti-CD5, -CD19, -CXCR4; for isolation of Ig subpopulations defined by IG densities, each sample was stained with anti-CD5, -CD19 and either anti-IgM or anti-IgD.

Gating strategy for FACS and phenotype measurements using FC. For CXCR4 and CD5 relative density fractions, $CD5^{+}CD19^{+}$ cells were gated, plotted for CXCR4 (Y axis) and CD5 (X axis), and subgated for the PF ($CXCR4^{Dim}CD5^{Bright}$), DDF ($CXCR4^{Dim}CD5^{Dim}$), IF ($CXCR4^{Int}CD5^{Int}$), DBF

(CXCR4^{Bright}/CD5^{Bright}), RF (CXCR4^{Bright}/CD5^{Dim}). Gating areas for each CXCR4/CD5 intraclonal fraction were adjusted in a sample-specific fashion to include ≤ 5% of total CD5$^+$CD19$^+$ in each gate. IF gating was based on the highest density of events (bulk of the clone). For the measurement of ^{2}H-DNA, 200,000 cells/subpopulation were sorted.

For intraclonal fractions based on the densities of smIgM or smIgD, CD5$^+$CD19$^+$ cells were gated and plotted for SSC (Y axis) and mean fluorescence intensity (MFI) after staining with soluble anti-IgM (a-IgM) or anti-IgD (a-IgD) (X axis) before subgating based on smIG fluorescence intensity; the latter defined Dim (IG^{Dim}), Intermediate (IG^{Int}), and Bright (IG^{Bright}) fractions. As for the CXCR4/CD5 fractions, gating area was adjusted in a sample-specific fashion to include ≤ 5% of total CD5$^+$CD19$^+$ in each gate. Int gating was based on the highest density of events (bulk of the clone). For those cases with two distinct bulk populations, Int gate was positioned at a relative equal distance from Dim and Bright gates. Mean fluorescence intensity (MFI) was used to infer the membrane density of the markers studied in the various intraclonal fractions. For the measurement of ^{2}H-DNA, 200,000 cells from each sorted fraction were used.

Measurement of deuterium incorporation in DNA. All fractions, stained and gated as described above, were sorted, pelleted, and flash frozen for subsequent genomic DNA extraction and mass spectrometry analysis. Complete methodology for deuterium quantification in newly synthesized DNA was performed as described. [1,24]

Cell size and phenotype measurement using IFC. Cells were automatically imaged in flow using an Amnis ImageStream X MKII (EMD Millipore) at 60X magnification. For this study, up to 5 channels were used: Ch1 = Brightfield; Ch2 = anti-IGs-FITC; Ch3 = anti-CD5-PE; Ch7 = anti-

CD19-Pacific Blue; Ch12 =SSC. Analysis for cell size and IG membrane densities were carried out as previously described. [16]

Stimulation through TLR9 and BCR. CLL B cells were cultured in an enriched medium consisting of RPMI1640 supplemented with insulin/transferrin/selenium supplement (BioWhittaker; cat # 17-8387), 2-ME (5×10^{-5} M) and 10% FCS. 10^5 cells were used in each culture. For TLR9 stimulation, CpG DNA TLR-9 ligand (ODN-2006; Invivogen) and recombinant human IL-15 (R&D Systems) were added at final concentrations of 15 ng/ml and 1.8 µM, respectively. For BCR signaling, polyclonal goat $F(ab')_2$ anti-IgM (Southern Biotech) and/or anti-IgD (Southern Biotech) were used at a final culture concentration of 20 µg/ml, and recombinant human IL-4 (Peprotech) was added at a final concentration of 20 ng/ml. For comparison of individual TLR9 and BCR stimulations, cells were cultured for 5 days alone (CTRL) or with ODN 2006 or with anti-IgM or anti-IgD. For chronological experiments, cells were cultured for 5 days alone (CTRL) or with ODN 2006 and anti-IgM or anti-IgD, either individually or together at Day 0, or one at Day 0 and the other at Day 3.

Supplementary References

1 Calissano, C.; Damle, R.N.; Hayes, G.; Murphy, E.J.; Hellerstein, M.K.; Moreno, C.; Sison, C.; Kaufman, M.S.; Kolitz, J.E.; Allen, S.L.; et al. In Vivo Intraclonal and Interclonal Kinetic Heterogeneity in B-Cell Chronic Lymphocytic Leukemia. Blood 2009, 114, 4832–4842, doi:10.1182/blood-2009-05-219634.

16 Mazzarello, A.N.; Gentner-Göbel, E.; Dühren-von Minden, M.; Tarasenko, T.N.; Nicolò, A.; Ferrer, G.; Vergani, S.; Liu, Y.; Bagnara, D.; Rai, K.R.; et al. B Cell Receptor Isotypes Differentially Associate with Cell Signaling, Kinetics, and Outcome in Chronic Lymphocytic Leukemia. Journal of Clinical Investigation 2022, 132, doi:10.1172/JCI149308..

24 Mazzarello, A.N.; Fitch, M.; Hellerstein, M.K.; Chiorazzi, N. Measurement of Leukemic B-Cell Growth Kinetics in Patients with Chronic Lymphocytic Leukemia. Methods Mol Biol 2019, 1881, 129–151, doi:10.1007/978-1-4939-8876-1_11.
